# Sensitivity to Morphine Reward Associates With Gut Dysbiosis in Rats With Morphine-Induced Conditioned Place Preference

**DOI:** 10.3389/fpsyt.2020.00631

**Published:** 2020-08-28

**Authors:** Jingyuan Zhang, Jun Yang, Cheng Yang, Ti Chen, Ziwei Wang, Junyi Li, Fanglin Qin, Qijian Deng, Xiaojie Zhang

**Affiliations:** ^1^ Department of Psychiatry, The Second Xiangya Hospital, Central South University, Changsha, China; ^2^ National Clinic Research Center for Mental Disorders, Changsha, China; ^3^ National Technology Institute on Mental Disorders, Changsha, China; ^4^ Hunan Key Laboratory of Psychiatry and Mental Health, Changsha, China; ^5^ Mental Health Institute of Central South University, Changsha, China; ^6^ Clinical Laboratory, The Third Xiangya Hospital, Central South University, Changsha, China

**Keywords:** morphine, gut microbiota, gut dysbiosis, conditioned place preference, sensitivity to morphine reward, rat model

## Abstract

Gut microbiota has been found to establish a bidirectional relationship with the central nervous system. Variations of the gut microbiota has been implicated in various mental disorders, including opioid use disorders. Morphine exposure has been repeatedly found to disrupt the gut microbiota, but association between the gut microbiota and the sensitivity to morphine reward remains unknown. In this study the conditioned place preference (CPP) paradigm was used for morphine-treated rats and saline-treated rats. After the CPP procedure, the morphine-treated rats were divided equally into the low and high CPP (L- and H-CPP) groups according to the CPP scores. We adopted 16S rRNA sequencing for the fecal bacterial communities at baseline and post-conditioning. By comparing the morphine-treated group with saline-treated group, we found alterations of microbial composition in the morphine-treated group, but no significant differences in alpha diversity. The L-CPP group and H-CPP group differed in microbial composition both before and after morphine treatment. The relative abundance of certain taxa was correlated to the CPP scores, such as *Alloprevotella* and *Romboutsia*. This study provides direct evidence that morphine exposure alters the composition of the gut microbiota in rats and that microbial alterations are correlated to the sensitivity to morphine reward. These findings may help develop novel therapeutic and preventive strategies for opioid use disorder.

## Introduction

An estimate of over 10^14^ microorganisms, including over 1000 species and over 7000 strains, inhabit the human gastrointestinal (GI) tract, the quantity of which is far more than all other cells in human bodies (1). The composition of this mass microbiota can be affected by many contributors, such as genetic factors (2), age (3), diet and exercise (4), etc. Gut microbiota has been found to establish a bidirectional relationship with the central nervous system, referred to as the gut-brain axis, possibly through immune, neural and endocrine pathways (5, 6). The variation of gut microbiota was demonstrated to be associated with a wide range of central nervous system disorders, including autism, anxiety, depression, and substance use disorders (7–9).

Morphine is a widely prescribed analgesic used for moderate to severe pain management, but its clinical use is impacted by side effects such as dependence and GI symptoms (10), including anorexia, constipation, nausea, vomiting, and bloating (11). Chronic morphine use could impair GI function and epithelial integrity (12–14). In humans, cirrhotic patients with long-term opioid use showed significant dysbiosis of gut microbiota, compared to cirrhotic patients without opioid use, especially, hepatic encephalopathy patients with chronic opioid use showed lower relative abundance of autochthonous taxa and *Bacteroidaceae* (15). In animals, after a slow release morphine pellet was implanted subcutaneously, mice with poly-microbial sepsis appeared elevated mostly in the *Firmicutes* phylum and Gram-positive bacterial species, and these disturbances were antagonized by the opioid receptor antagonist naltrexone (16). Evidence also showed that chronic morphine treatment significantly caused dysbiosis and preferential expansion of Gram-positive bacteria, and reduction in bile-deconjugating bacterial strains (17). Morphine-induced gut dysbiosis in mice exhibited a significant increase in communities related to pathogenic function and a decrease in communities related to stress tolerance (18). Unique alterations in gut microbiota composition were noted following intermittent or sustained morphine administration (19). The above mentioned studies provided a basis for the effect of morphine use on gut microbiota.

However, these studies mainly concentrated on the gut microbial comparison between the morphine group and control group to reveal the association between morphine treatment and gut microbiota; but ignored the influence of individual differences within each group. In fact, individuals report a variety of analgesia to the same doses of opioids, and not all opiate users develop addiction clinically (20). Certain individuals are more vulnerable to addiction than others because of higher sensitivity to drug effects. This individual sensitivity to substance could be affected by genetic, molecular and behavioral contributors (21). Drawing on discoveries in association between gut microbiota and substance use disorders, the role of gut microbiota in the sensitivity to the substance reward is worth further study. Researchers have adopted rat models of alcohol self-administration and divided models into vulnerable and resilient groups. The vulnerable group had increased impulsive and compulsive behaviors, and increased relapse after abstinence, and the taxa of gut microbiota presented a change in trend compared to the resilient group (22). With the treatment of non-absorbable antibiotics, animals showed reduced gut microbiota and enhanced sensitivity to cocaine reward and locomotor-sensitizing effects of repeated cocaine administration (23). Antibiotic treatment reduced opioid analgesic potency and impaired cocaine reward in morphine dependent models (19). These studies provided direct evidence of association between gut microbiota and rodents’ sensitivity to alcohol or cocaine reward. It is well established that although various substances have different pharmacological actions, their final common pathway is to act on the midbrain limbic dopamine (DA) system, the center of reward system and promote DA neuron impulses and DA release (24). Therefore, we suspect that gut microbiota may also play a role in morphine reward.

Conditioned place preference (CPP) paradigm is one of the most popular measures of “reward” in rodents. During the paradigm, the rewarding property of a drug is assessed by its ability to promote the formation of a preference for an environment in which the drug has been repeatedly treated (25).The CPP scores could reflect the sensitivity to rewarding values of the drug (26, 27).

Our goal was twofold. First, we aimed to establish morphine-induced conditioned place preference (CPP) rat models, and adopted 16S rRNA sequencing of fecal bacterial communities before and after morphine exposure, with an attempt to describe the impact of morphine on the alterations in gut microbiota. Second, we compared the gut microbiota of rats with different CPP scores and examined the correlation between microbial alterations and the sensitivity to morphine reward.

## Materials and Methods

### Animals

Male Sprague-Dawley rats were purchased from Hunan Silaike Jingda Laboratory Animal, China. Their body weight ranged approximately between 180g to 200g. Each cage housed four rats. The temperature (20°C~25°C), humidity (50%~55%) and light/dark cycle (12h/12h) were controlled. The rats were offered enough food and water. All animals were maintained in pathogen-free facilities. The experimental protocol was approved by the Ethics Committee of the Second Xiangya Hospital of Central South University (ethical approval no. 2015-063).

### Chemicals and Drugs

Morphine was purchased from Northeast Pharmaceutical group Shenyang NO.1 Pharmaceutical Co., Ltd. It was diluted in 0.9% sterile saline.

### Conditional Place Preference

The CPP apparatus consisted of two larger compartments (60cm L × 40cm W × 40cm H) and one connection zone (40cm L × 6.5cm W × 60cm H), and the connection zone served as a connection between the two compartments. Each compartment was separated by an optional manual guillotine door. One floor had horizontal black and white stripes with metal bars, and the other had vertical black and white stripes with wire-mesh, providing the visual-cue difference between the two larger compartments. A video camera, transmitting the data to the analysis software (Jiliang Software and Instruments, Shanghai, China), was above the apparatus which recorded the locomotor activity of rats and the time they spent in each compartment. The apparatus was illuminated with two spotlights mounted on either side of the camera. The CPP procedure was assigned into four phases: habituation, preconditioning, conditioning and post-conditioning.

All the Sprague-Dawley rats were randomly divided into two groups: morphine group (n=28) and saline group (n=7). For convenience, we named morphine group at baseline as the MB group, morphine group for post-treatment as the MP group, saline group at baseline as the SB group, saline group for post-treatment as the SP group. All the rats lived with unlimited food and water intake. Rats adapted to the environment for three days (day1, 2, 3). We initially placed the rats into the connection zone of the CPP apparatus with the door removed for 30 minutes to adapt to the apparatus and return it to the home cage (day4). The next day rats were placed into the connection zone with the door removed for 15 minutes to test for preference at baseline (day5). When we divided rats in the morphine group into two groups according to CPP scores, the rats either stayed in the connecting zone more than 70% of time or passed through the guillotine door less than ten times, or both at baseline were removed. Here we removed four rats in morphine group. During the conditioning phase, the rats were injected with morphine (0.9%; 10mg/ml, 1ml/kg, i.p. the morphine group) or equal saline (0.9%, 1ml/kg, i.p. the saline group) and confined to the non-preference side (morphine-paired side) for 15 minutes (day6, 8, 10, 12). On alternate days, all rats were injected with equal saline (0.9%, 1ml/kg, i.p.) and confined to the preference side for 15 minutes (day7, 9, 11, 13). The morphine group has four-cycle conditioning training. During the Post-conditioning phase (day14), the rats were allowed free access to the two compartments to test preference after treatment. The video camera and the CPP software recorded the time they spent in the bilateral compartments and locomotor activity of rats throughout Preconditioning and Post-conditioning phase. The CPP score means the difference between time spent in the drug-paired side after CPP training and time at baseline (28). The remaining 24 rats of the morphine group were divided into two groups according to the CPP score, one group of 12 rats with higher CPP scores was designated as the H-CPP group, while the other group of 12 rats with lower CPP scores was designated as the L-CPP group.

### Fecal Sample Collection

Fecal samples were freshly collected at day 5 (Preconditioning) and day 14 (Post-conditioning) and stored at-80C until use.

### DNA Extraction and PCR Amplification

E.Z.N.A.^®^ Stool DNA Kit (Omega Bio-tek, Norcross, GA, U.S.) was used to extract fecal microbial DNA from the fecal sample according to the manufacturer’s instructions. The bacterial genomic DNA was amplified with 341F (5’-barcode-CCTACGGGNGGCWGCAG-3’) and 805R (5’-GACTACHVGGGTATCTAATCC’) specific for the V3 and V4 region of 16S rRNA, where the barcode was an eight-base sequence unique to each sample. PCR reactions were performed in triplicate 20 μL mixture containing adequate TopTaq DNA Polymerase kit (Transgen, China) and 10 ng of the template DNA using the 2720 Thermal Cycler (Thermo Fisher Scientific, USA) and setting the cycling parameters (94°C for 3 min, followed by 25 cycles at 94°C for 10s, 55°C for 15s, and 72°C for 30 s and a final extension at 72°C for 7 min).

### Illumina MiSeq 16S rRNA Sequencing

We purified amplicons with AgencourtAMPureXPPCR Purification Beads (Beckman Coulter, USA) according to the manufacturer’s instruction. Amplicons were quantified using Invitrogen Qubit3.0 Spectrophotometer (Thermo Fisher Scientific, USA) and Agilent 2100 Bioanalyzer (Agilent Technologies, USA). A master DNA pool was generated from the purified products in equimolar ratios. Purified amplicons from the samples were sent out for pyrosequencing on an Illumina MiSeq platform at Gensky Biotechnology (Shanghai, China).

### Processing of Sequencing Data

All sequences in the FASTQ file format were filtered and merged following three criteria: (i) Using TrimGalore to truncate raw reads whose average quality are lower than 20, deleting the sequences that contain the adapter (ii) Exact barcode matching, two nucleotide mismatches in primer matching and reads containing ambiguous characters and primers were removed. (iii) Sequence assembling, using FLASH (v1.2.11) to assemble the paired end reads, reads that could not be assembled were discarded.

After filtering, the sequences were matched at the operational taxonomic unit (OTU) using UPARSE (version 7.1). OTU clustering was performed with a 97% similarity cut-off. Chimeric sequences were identified and removed using UCHIME. Taxonomic assignments were determined by Mothur with Ribosomal Database Project (RDP) (29), SLIVA 16S rRNA database (30) and UNITE database (31) set with a confidence threshold of 80%. All bacteria at family and genus levels analyzed were shown in **Table S1**.

### Statistical Analysis

We used Mothur (version v.1.30.1 4) to calculate and analyze microbial community estimators, including richness estimators (the ACE index and Chao index) and alpha diversity estimators (the Simpson index and Shannon index). Rarefaction curve was generated for the observed OTU. All correlations were performed by Spearman correlation for data in accordance with bivariate normal distribution or Pearson correlation for data not in accordance with bivariate normal distribution. A Mann-Whitney test, Wilcoxon signed rank test and Student’s t test were used to determine whether differences of median existed. An F test was assessed for equal variance, and normality was assessed by the Shapiro-Wilk normality test. All tests were two-sided. The statistical significance threshold was defined as *p*-value <0.05. Besides, when we compared the micrbiota of saline group with that of morphine group and made rarefarction curve, the whole 28 rats in morphine group were included in the analysis. While we compared the microbiota of H-CPP withe that of L-CPP group, four rats had been removed and the remaining 24 rats were included in analysis.

## Results

### Morphine-Induced CPP Model Was Established and Rats Presented Individual Differences in Sensitivity to Morphine Reward

**Figure 1:**
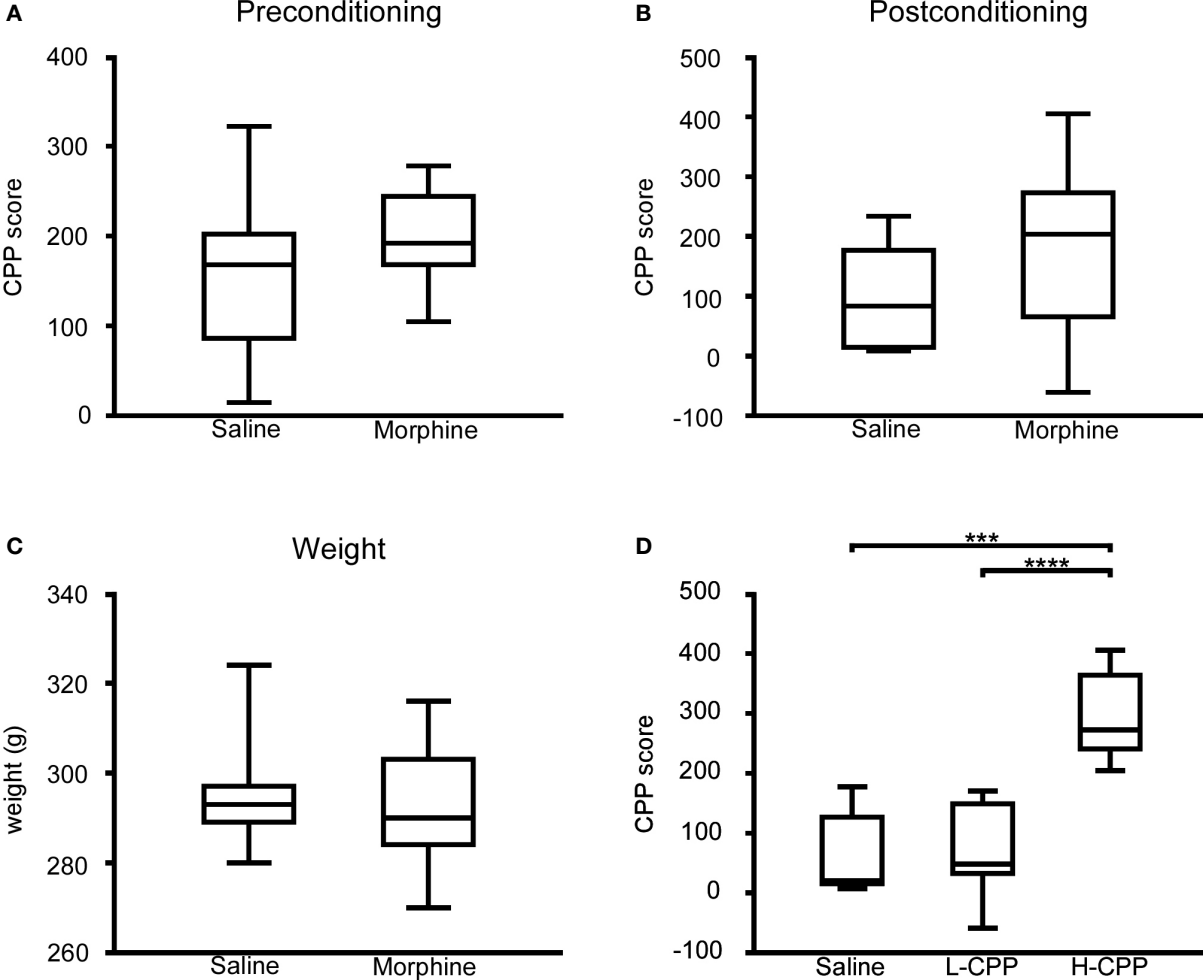
**(A)** The conditional place preference (CPP) score of rats were compared between the morphine and saline group in the preconditioning phase, and there was slight difference appeared in the CPP score between the morphine group and saline group. **(B)** The CPP score of rats were compared between the morphine and saline group in the postconditioning phase, and there was slight difference appeared in the CPP score between the morphine group and saline group. **(C)** The weights of rats were compared between the morphine and saline group in the preconditioning phase. There was no significant difference between the weight of posttreatment for morphine and saline group. **(D)** The CPP score of rats were compared among the saline, L-CPP and H-CPP group in the postconditioning phase. The CPP score of H-CPP is significantly higher than L-CPP and saline group, and there was no difference between the CPP score of L-CPP and saline group. The CPP score means the difference between time spent in the drug-paired side after CPP training and time at baselinSe.t udent’s t test was used to analyze the data. The central line shown in each box plot indicates the median of data. Whiskers extend to cover the whole range of values. Statistical significance was accepted at p<0.05. *p<0.05; **p<0.01; ***p<0.001; ****p<0.0001.

During the preconditioning phase, as we expected, there was no significant difference in the CPP scores between the morphine group and saline group (p=0.1616; **Figure 1A**). After the morphine or saline treatment, the CPP score of the morphine group was slightly higher than the saline’s (p=0.0783; **Figure 1B**). According to this result, the rats in the morphine group were exposed to morphine. After the four-cycle conditioning training, the weights of rats all increased, but there was no difference in weight between the MP group and SP group (p=0.4587; **Figure 1C**).

Although rats in the morphine group were exposed to the same dose of morphine, rats presented individual differences in the CPP score. The CPP score served as a surrogate of rats’ sensitivity to morphine, which can evaluate the rewarding and reinforcing properties of morphine (26). The H-CPP group showed a significant increase in the CPP scores compared to the L-CPP and SP (p<0.0001 for H-CPP versus L-CPP; p=0.0004 for H-CPP versus SP; **Figure 1D**), which implicated that the rats in the H-CPP group had prominent preference and higher sensitivity to morphine than L-CPP. The L-CPP group showed no significant difference compared to the SP (p=0.8229; **Figure 1D**).

### Morphine Altered the Gut Microbiota Composition Pattern, but Not the Alpha Diversity of the Microbial Community

**Figure 2:**
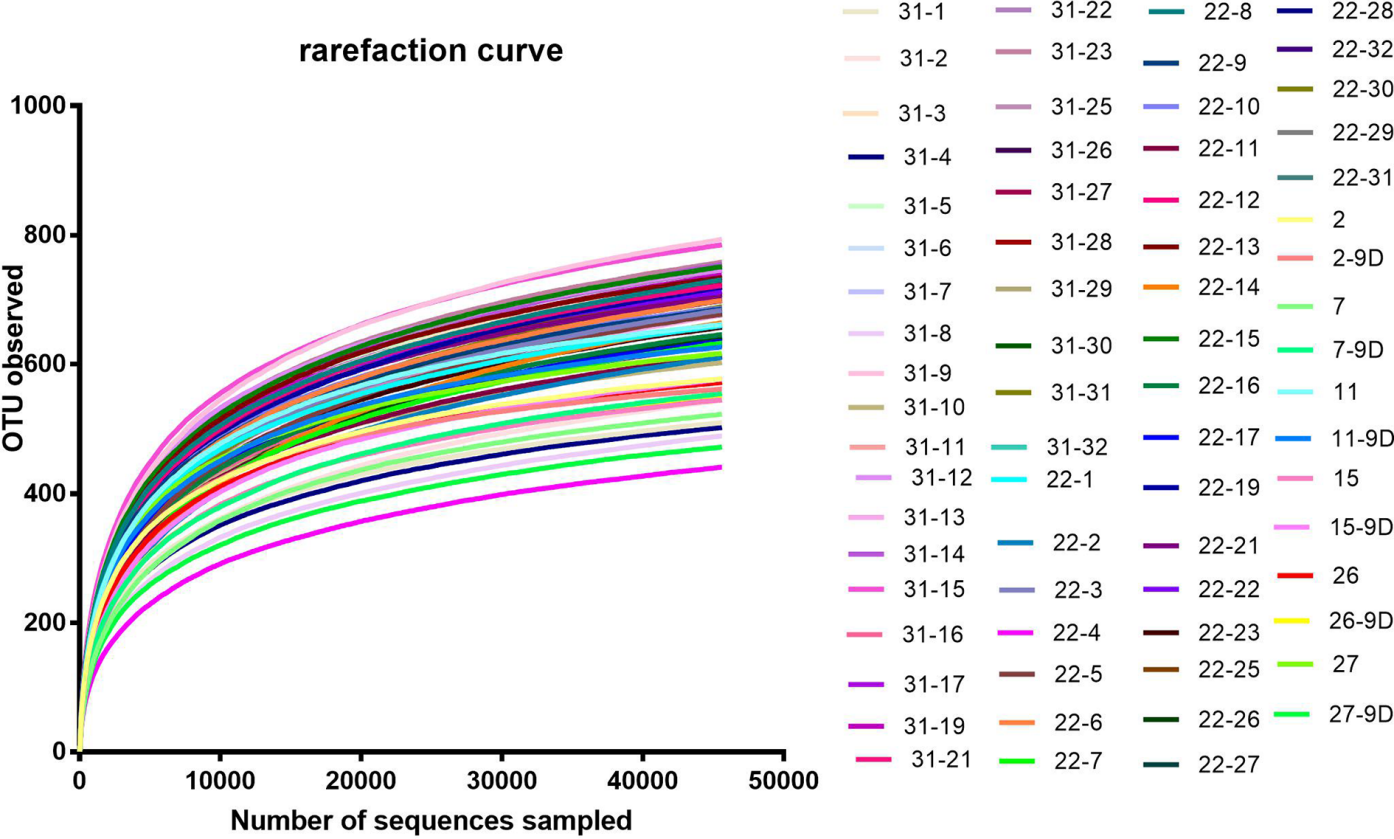
Rarefaction curve. Rarefaction curves were based on the 16S rRNA gene sequencing of the all samples from baseline group and the post-treatment for morphine and saline group. The rarefaction curve suggested that the sequencing depth was satisfactory and represented the majority of bacterial species, because the curves became relatively flat as the number of sequences analyzed increased.

**Figure 3:**
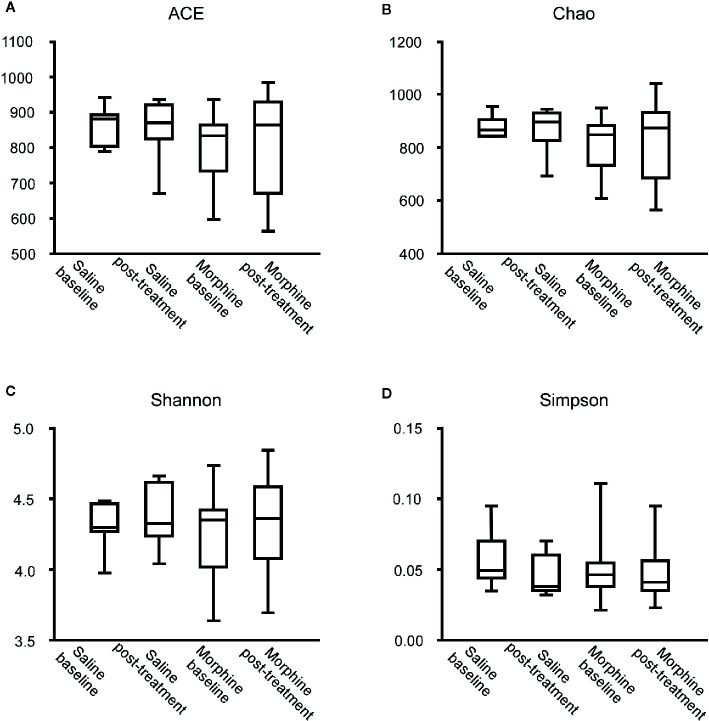
Comparison of the community alpha diversity. Alpha diversity was measured by ACE **(A)**, Chao **(B)**, Shannon **(C)** and Simpson **(D)**. A Mann-Whitney test or student’s t test was used to analyze the data. The central line shown in each box plot indicates the median of data. Whiskers extend to cover the whole range of values. Statistical significance was accepted at p < 0.05. *p < 0.05; **p < 0.01; ***p < 0.001; ****p < 0.0001.

According to the rarefaction curve (**Figure 2**), the sequencing depth was satisfactory and represented the majority of bacterial species. We evaluated ecological features of fecal bacterial communities using several indices based on the OTU level. The richness estimators, such as the ACE index and Chao index, were used to extrapolate species richness. The diversity estimators, such as the Shannon index and Simpson index, were used to extrapolate species richness and evenness. The ACE indexes (mean ± SD) were 867.4 ± 54.4, 857.6 ± 92.16, 801.6 ± 99.92 and 811.5 ± 133 for the SB, SP, MB and MP groups, respectively. Chao indexes were 886.8 ± 42.31, 869.4 ± 88.05, 815.6 ± 102.6 and 821.5 ± 136.3, Shannon were 4.301 ± 0.1692, 4.384 ± 0.2279, 4.243 ± 0.2999 and 4.295 ± 0.3134, and Simpson were 0.5655 ± 0.0202, 0.04586 ± 0.01522, 0.05043 ± 0.02189 and 0.04935 ± 0.02056 for the SB, SP, MB and MP groups, respectively (**Figures 3A–D**). These four indexes ACE, Chao, Shannon, and Simpson all showed no significant differences among the SB, SP, MB and MP groups. We also compared alpha diversity between L-CPP and H-CPP both at baseline and after treatment. It was found that alpha diversity did not appear significantly different in these comparisons (**Figures S1** and **S2**).

**Figure 4:**
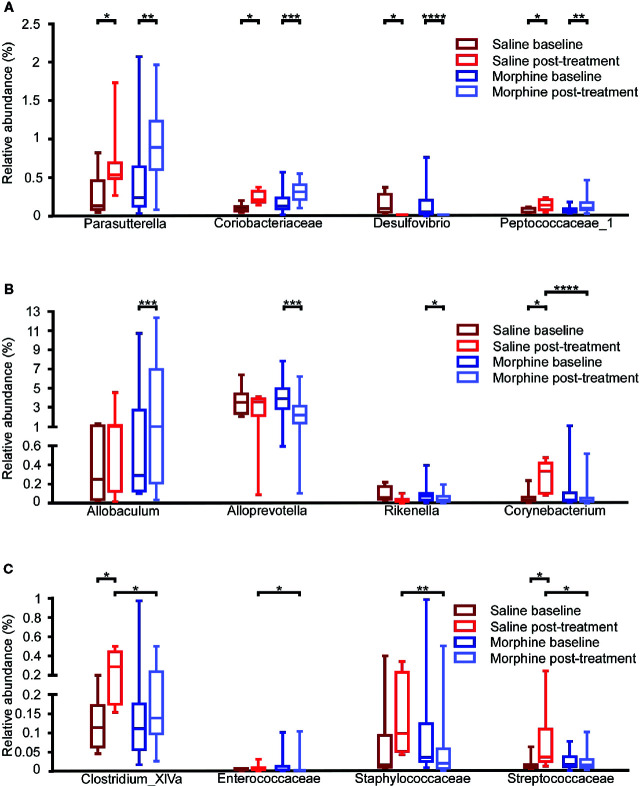
Comparison of the gut microbiota composition among the baseline and post-treatment for the morphine and saline group. Comparison of the relative abundance of gut microbiota at the family and genus levels among the saline-baseline, saline post-treatment, morphine-baseline and morphine post-treatment groups. **(A)** The relative abundance of *Parasutterella*, *Coriobacteriaceae*, *Desulfovibrio* and *Peptococcaceae_1* were shown. **(B)** The relative abundance of *Allobaculum*, *Alloprevotella*, *Rikenella* and *Corynebacterium* were shown. **(C)** The relative abundance of *Clostridium_XlVa*, *Enterococcaceae*, *Staphylococcaceae* and *Streptococcaceae* were shown. A Mann-Whitney test and Wilcoxon signed rank test were used to analyze the data. A student’s t test was used for the data with Gaussian distribution. The central line shown in each box plot indicates the median of data. Whiskers extend to cover the whole range of values.Statistical significance was accepted at p < 0.05. *p < 0.05; **p < 0.01; ***p < 0.001; ****p < 0.0001.

To assess whether morphine altered the gut microbial community, we compared the gut microbiota between the baseline and post-treatment for the morphine and saline group respectively (**Figures 4A–C**). At the genus level, when compared the MP to MB, *Allobaculum* (p=0.0002) and *Parasutterella* (p=0.0061) were more abundant, and *Alloprevotella* (p=0.0002), *Desulfovibrio* (p<0.0001) and *Rikenella* (p=0.0176) were less abundant in the MP. In the saline group, *Clostridium_XlVa* (p=0.0125), *Corynebacterium* (p=0.0156), and *Parasutterella* (p=0.0313) increased and *Desulfovibrio* (p=0.0156) decreased after saline treatment. At the family level, when compared the MP to MB, *Coriobacteriaceae* (p=0.0004) *and Peptococcaceae_1* (p=0.0034) were more abundant in the MP. In the saline group, *Coriobacteriaceae* (p=0.0181), *Peptococcaceae_1* (p=0.0156) and *Streptococcaceae* (p=0.0156) increased after saline treatment.

Additionally, we compared the MP to SP. It was found that the MP had lower relative abundance of *Corynebacterium* (p<0.0001), and *Clostridium_XlVa* (p=0.0117) at the genus level, *Enterococcaceae* (p=0.0325), *Staphylococcaceae* (p=0.0035), *Streptococcaceae* (p=0.0201) at the family level. All the taxa with significance are shown in **Table S2** and **Table S3**.

### Differences in the Microbial Composition Between H-CPP and L-CPP After The Morphine-Induced CPP Training

**Figure 5:**
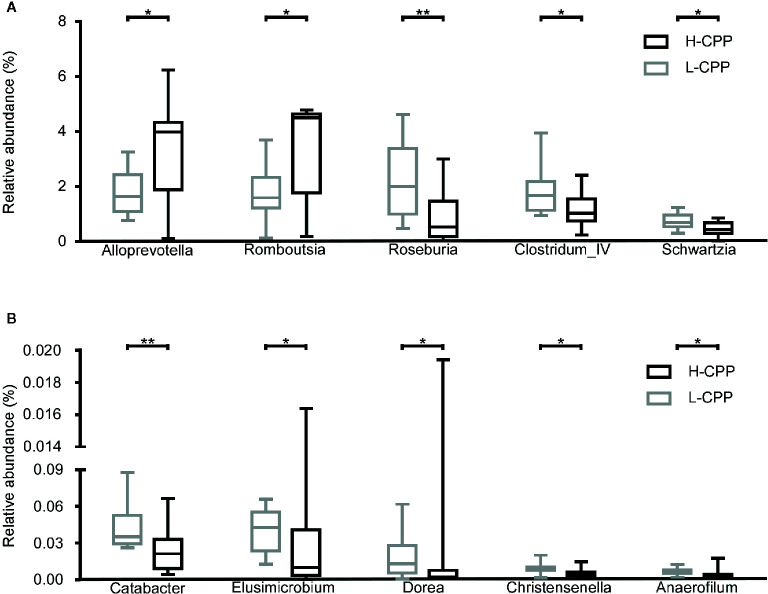
Comparison of the gut microbiota composition between the low and high conditional place preference group (L-CPP versus H-CPP) after the morphine-induced CPP training. **(A)** The relative abundance of *Alloprevotella*, *Romboutsia*, *Roseburia*, *Clostridium_IV* and *Schwartzia*, were found to be different between L-CPP and HCPP. **(B)** The relative abundance of *Catabacter*, *Elusimmicrobium*, *Dorea*, *Christensenella* and *Anaeroflium* were found to be different between L-CPP and H-CPP. A Mann-Whitney test was used to analyze the data. The central line shown in each box plot indicates the median of data. Whiskers extend to cover the whole range of values. A student’s t test was used for the data with Gaussian distribution. Statistical significance was accepted at p < 0.05. *p < 0.05; **p < 0.01; ***p < 0.001; ****p < 0.0001.

In order to find the taxa that were associated with morphine preference, we compared microbiota in H-CPP to L-CPP. At the family level, the following bacterial decreased: *Spirochaetaceae* (p=0.0252), *Veillonellaceae* (p=0.0236), *Catabacteriaceae* (p=0.0087), *Elusimicrobiaceae* (p=0.0196) and *Christensenellaceae* (p=0.0474), while *Peptostreptococcaceae* (p=0.01) increased in the H-CPP group. At the genus level, *Alloprevotella* (p=0.0384) and *Romboutsia* (p=0.01) increased, whereas *Clostridium_IV* (p=0.0387), *Roseburia* (p=0.0036), *Schwartzia* (p=0.0236), *Catabacter* (p=0.0087), *Elusimicrobium* (p=0.0196), *Dorea* (p=0.0423), *Christensenella* (p=0.0471) and *Anaerofilum* (p=0.0185) decreased significantly in the H-CPP group (**Figures 5A, B**). All the taxa at the family and genus levels with significance are shown in **Table S4**.

**Figure 6:**
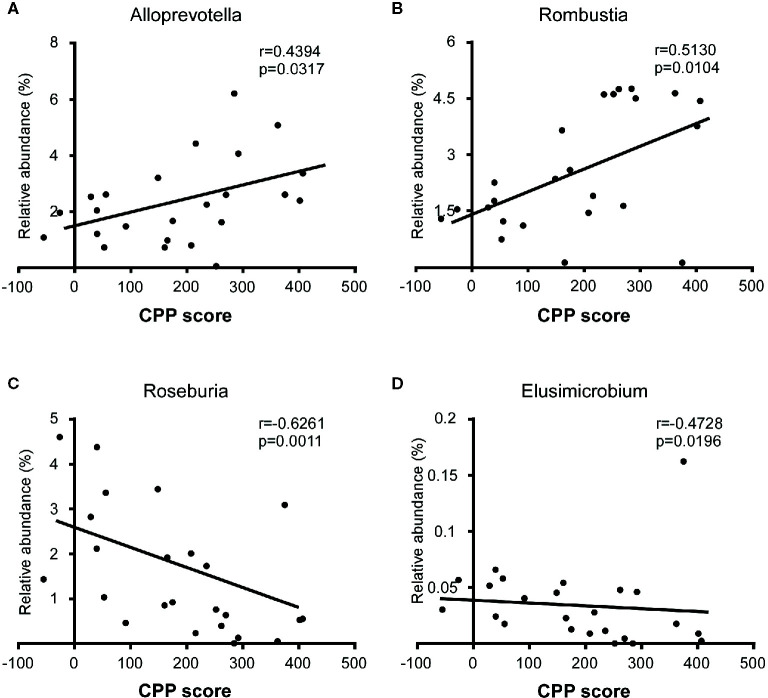
Correlation between specific taxa and the CPP scores after the morphine-induced CPP training. The relative abundance of *Alloprevotella* **(A)** and *Romboutsia* **(B)** were positively correlated with the CPP score, while the relative abundance of *Roseburia* **(C)** and *Elusimicrobium* **(D)** were negatively correlated with the CPP score. A Spearman correlation was performed for *Romboutsia*, *Roseburia* and *Elusimicrobium*, and a Pearson correlation was performed for *Alloprevotella*. The r value and p value were used to evaluate statistical significance.

To identify whether microbial taxa correlated with the CPP score, we performed Pearson correlation or Spearman correlation between the relative abundance of bacterial and the CPP scores. At the family level, we found that the relative abundance of *Lachnospiraceae* (r=-0.4946, p=0.0140), *Ruminococcaceae* (r=-0.5681, p=0.0038) and *Elusimicrobiaceae* (r=-0.4728, p=0.0196) were negatively correlated with the CPP scores. At the genus level, the relative abundance of *Alloprevotella* (r=0.4394, p=0.0317) and *Romboutsia* (r=0.5130, p=0.0104) were positively correlated with the CPP scores, while the relative abundance of *Roseburia* (r=-0.6261, p=0.0011) and *Elusimicrobium* (r=-0.4728, p=0.0196) were negatively correlated with the CPP scores (**Figures 6A–D**).

### Differences in the Microbial Composition Between L-CPP and H-CPP at Baseline

**Figure 7:**
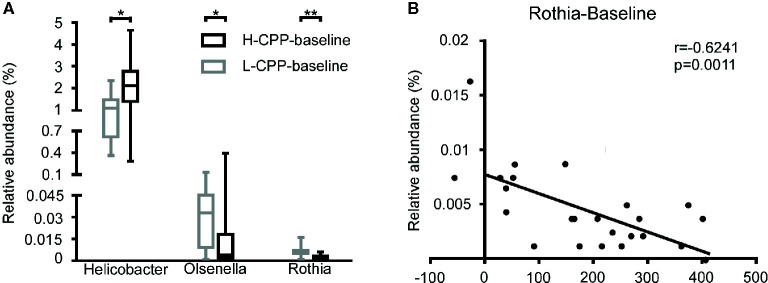
Comparison of the gut microbiota composition between L-CPP and H-CPP groups at baseline and the correlation between the relative abundance of *Rothia* at baseline and CPP score. **(A)** The significant differences among *Helicobacter*, *Olsenella*, and *Rothia* were noted. A Mann-Whitney test was used to analyze the data. A Wilcoxon signed rank test was used to analyzed the data. A student’s t test was used for data with Gaussian distribution. The central line shown in each box plot indicates the median of data. Whiskers extend to cover the whole range of values. Statistical significance was accepted at p<0.05. **(B)** The relative abundance of *Rothia* was negatively correlated with the CPP score. Spearman correlation was performed for *Rothia*. The r value and p value were used to evaluate statistical significance. *p<0.05; **p<0.01; ***p<0.001; ****p<0.0001.

After analyzing the differences in the microbial composition between H-CPP and L-CPP, we hypothesized that some specific bacteria might determine the sensitivity to morphine before the morphine administration. We analyzed the bacterial composition at baseline between H-CPP group and L-CPP group. At the family level, in H-CPP-baseline, *Helicobacteraceae* (p=0.0238) was more abundant, while *Puniceicoccaceae* (p=0.0237) was less abundant. At the genus level, the H-CPP-baseline appeared to have elevated *Helicobacte*r (p=0.0238), and decreased *Olsenella* (p=0.0496) and *Rothia* (p=0.0097; **Figure 7A**). We also performed the Spearman correlation between the relative abundance of bacteria at baseline and the CPP scores. We found the relative abundance of *Rothia* (r=-0.6241, p=0.0011; **Figure 7B**) was negatively correlated with the CPP score.

### Within-Group Differences in the Relative Abundance Between the Baseline and Post-Conditioning

We compared the within-group differences between the baseline and post-conditioning for the L-CPP and H-CPP group at the genus and family levels (**Table S5**, **S6**). According to the characteristics of results, we divided these taxa with significant differences into three types. In the first two types, the microbiota only showed significant difference in the L-CPP or H-CPP group. The last type was characterized by the microbiota with significant differences both in L-CPP and H-CPP groups.

## Discussion

Our study is the first to find that rats with different sensitivities to morphine reward also showed a varying composition of gut microbiota. In the morphine group, although the rats belonged to the same species, received the same additional manipulations, and fed in the same environment, the rats presented individual differences in the CPP scores (32). The CPP score was used to assess the sensitivity to morphine reward (26, 27). Comparing the microbiota of the H-CPP and L-CPP groups, we found different gut microbiota compositions both at baseline and after morphine treatment. Recent studies indicated that gut dysbiosis played an important role in the sensitivity to morphine effects. Antibiotic cocktail or probiotics treatment significantly reduced the morphine analgesic tolerance in morphine-treated mice (33, 34). Our results are the first to reveal the relationship between gut microbiota and the sensitivity to morphine reward.

We found that the relative abundance of specific bacterial taxa was in correlation to the CPP scores. Comparing the gut microbiota of H-CPP to L-CPP at baseline, we found significant difference in the *Olsenella*, *Rothia*, and *Helicobacter*. *Rothia* was negatively correlated with the CPP score. Intriguingly, researchers found that a maternal high-fat diet led to a shift of the offspring gut microbiota, including reduced *Olsenella* and *Helicobacter*, and this shift prevented long-lasting neural adaptation in the mesolimbic DA reward system (35). This result provided evidence for the association between these two taxa and the reward systems. Due to these differences showed before the morphine treatment, the differential relative abundance of these taxa may be the nature of rats with high/low sensitivity to morphine. These taxa may become the predictors of sensitivity to the morphine reward. Individuals with decreased *Olsenella* and *Rothia*, and increased *Helicobacter* may have a higher risk of addictive behaviors after morphine exposure. Additionally, researchers found that some common signal paths may be involved in the process of gut microbiota affecting the rewarding property of morphine. Gut microbial metabolites including short-chain fatty acids and bile acids putatively influence this process (9). Short-chain fatty acids, as histone deacetylase inhibitors, could contribute to the extinction of morphine-induced CPP (36). Reddy et al. conducted a bariatric surgery for mice to augment circulating bile acids, which appeared to decrease the rewarding properties of cocaine (37). Besides, microbial metabolites modulate production of inflammatory cytokines such as interferon-alpha and interleukin-10. Rats with interferon-alpha treatment showed potentiated reinstatement of the morphine CPP (38). However, interleukin-10 over expression reduced the morphine CPP scores and acquisition of self-administration (39, 40). Collectively, these results suggest that microbial metabolites are probably linked to morphine sensitivity.

We also found differences between the H-CPP and L-CPP groups after morphine treatment. The three taxa *Olsenella*, *Rothia* and *Helicobacter* which showed differences at baseline, showed no differences after the training. We speculated that because the H-CPP group was more sensitive to morphine reward, morphine exerted a stronger effect on gut microbiota in the H-CPP than the L-CPP group after the same morphine exposure. However, we found no significant difference in the alpha diversity of microbiota appearing both at baseline and post-treatment, which indicated that rats with different sensitivity to morphine reward showed no essential difference in richness and diversity of gut microbiota. To summarize, this study revealed the relationship between the composition of gut microbiota and sensitivity to the morphine reward. Given that sensitization to the drug effects is regarded as an important role in the development of drug addiction (41), our finding may provide basis for predicting the risk of addition and direct addiction treatment.

At last, we compared the MP with the SP in order to observe the morphine effect on microbiota. It is noted that some taxa changed both after morphine and saline treatment, including *Alistipes* and *Adlercreutzia* at genus level and *Coriobacteriaceae* and *Rikenellaceae* at family level. We guess that the additional manipulations mainly contributed to these alterations. The additional manipulations, such as grabbing, saline or morphine injection and even changed living environment, could become stimuli to rats and caused abnormality of physiology, pathological and mental state (8, 42). Several studies have described an increase in *Coriobacteriaceae* in rat models experiencing depression and stress (43–45). Maybe these stimuli become stressors, which can lead to stress or stress-related disorders (46). While other taxa changed in different ways after morphine or saline treatment, which may be induced by the morphine inherent effects. Prior studies showed that morphine itself could impair the GI function and epithelial integrity, and disrupt gut homeostasis (12–14). However, we found no significant difference in weight between the morphine group and saline group, so we ignored the possible effect of morphine on appetite and absorption. Anxiety, depression, and memory deficits are common adverse effects of morphine use or morphine addiction, whose relation with gut microbiota has been repeatedly identified (47–52). It is worth noting that morphine is a prescribed analgesic that can depress the central nervous system and induce sedative and antalgic actions (10). Thus morphine might offset some stress effects caused by additional manipulations, which caused the differences of some taxa in the comparison between the SB and SP but not in the comparison between MB and MP. Besides, the addictive property of morphine or the addiction-related behaviors might have an influence on gut microbiota. Our discovery was consistent with recent studies that found increased *Clostridium* and *Enterococcus* in morphine dependence models (17–19). Nevertheless, it is still uncertain that the above alterations in gut microbiota were caused by the effects of morphine itself or the addictive property.

This is the first study to discover that microbial alterations are connected to the sensitivity to morphine reward. Our study had several limitations. First, gut microbiota can be altered by many host factors, such as genes, physiological and pathological state and internal and external environment (53), which we did not control in the current study. Second, the causality between sensitivity to morphine reward and gut microbiota is not clear. Further studies on probiotics, antibiotics, and bacterial metabolites are needed to determine how manipulations of the microbiome may affect the rewarding effects of morphine. In this study, we only adopted rat models to prove the correlation between rewarding effects of morphine and gut microbiota, so the correlation in other species is to be determined. Specifically, although animal models allow for greater experimental control than human studies, there are great differences in microbiome compositions between humans and animal models, due to the large differences in size, metabolic rate and dietary habits (54). Additionally, it will be important to define whether gut dysbiosis responds to rewarding properties of other substance, like psychostimulants and alcohol. However, our findings do suggest gut dysbiosis may be a biomarker and a predictor to the risk of developing opioid use disorder. Gut microbiota may become a novel therapeutic target for opioid use disorder.

## Data Availability Statement

The datasets presented in this study can be found in online repositories. The names of the repository/repositories and accession number(s) can be found below: https://www.ncbi.nlm.nih.gov/, PRJNA 559598.

## Ethics Statement

The animal study was reviewed and approved by Ethics Committee of the Second Xiangya Hospital of Central South University (ethical approval no. 20150063).

## Author Contributions

XZ conceived and designed the experiments. JY, JZ, CY, JL, and FQ performed the experiments. JY and JZ analyzed data and contributed reagents, materials, and analysis tools. JY and JZ interpreted the results and wrote the paper. All authors contributed to the article and approved the submitted version.

## Funding

This work was supported by National Key R&D Program of China (2017YFC1310400) and National Natural Science Foundation of China (NSFC) Grant 81501108 for XZ, Central South University Undergraduate Research and Innovation (URI) Program (201810533044) for JY.

## Conflict of Interest

The authors declare that the research was conducted in the absence of any commercial or financial relationships that could be construed as a potential conflict of interest.
