# Supplementary material for: Sensitivity to Morphine Reward Associates With Gut Dysbiosis in Rats With Morphine-Induced Conditioned Place Preference
Source: Front Psychiatry. 2020 Aug 28;11:631. doi: 10.3389/fpsyt.2020.00631 (PMC7484999; doi:10.3389/fpsyt.2020.00631)
Supplement: Supplementary file 1 [file DataSheet_1.docx]

Supplementary Material

# Supplementary Figures


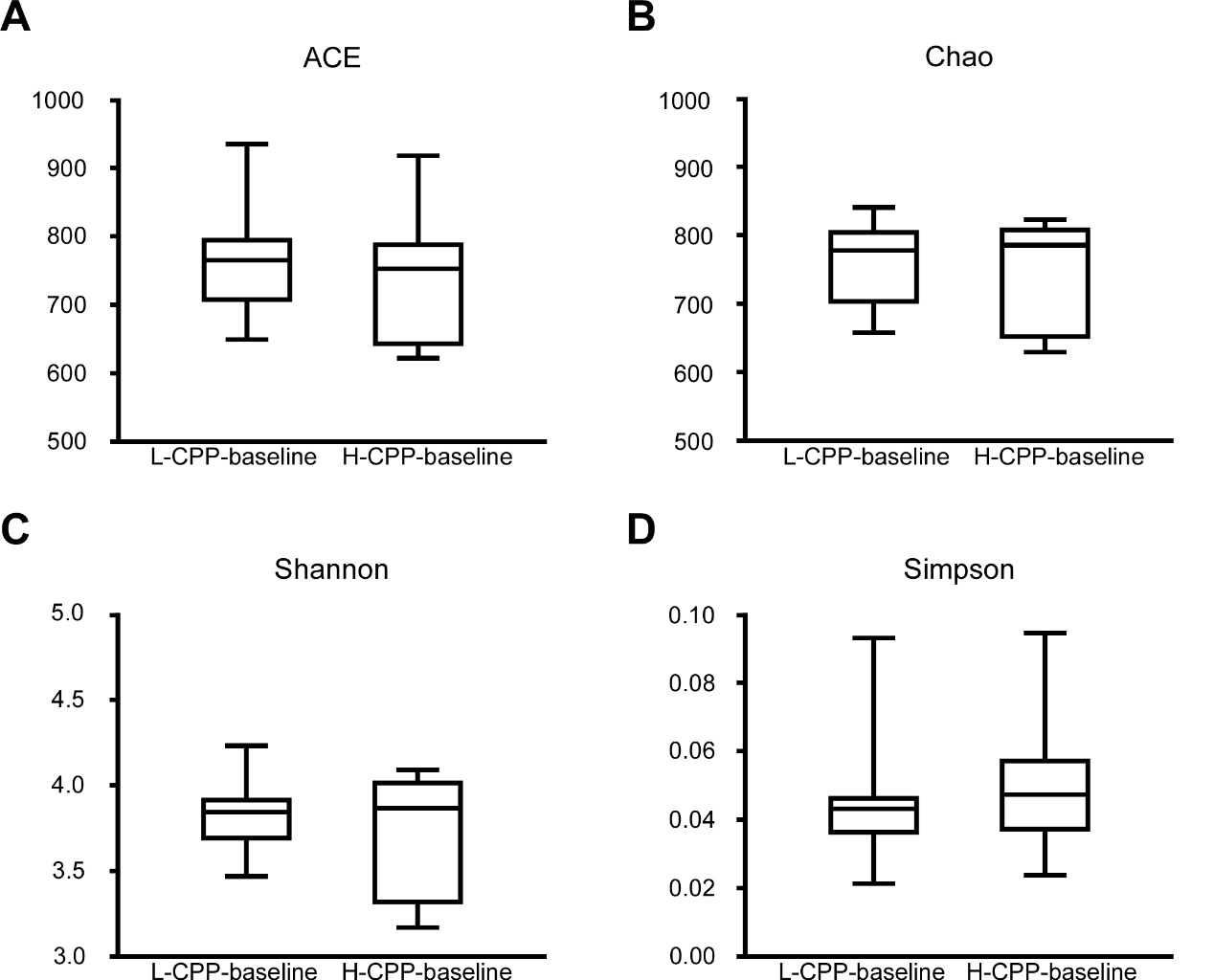


**Figure S1: Comparison of the community alpha diversity between L-CPP-baseline and H-CPP-baseline.** Alpha diversity was measured by ACE **(A)**, Chao **(B)**, Shannon **(C)** and Simpson **(D)**. A Mann-Whitney test was used to analyze the data. A student’s t test was used for the data with Gaussian distribution. The central line shown in each box plot indicates the median of data. Whiskers extend to cover the whole range of values. Statistical significance was accepted at p<0.05.


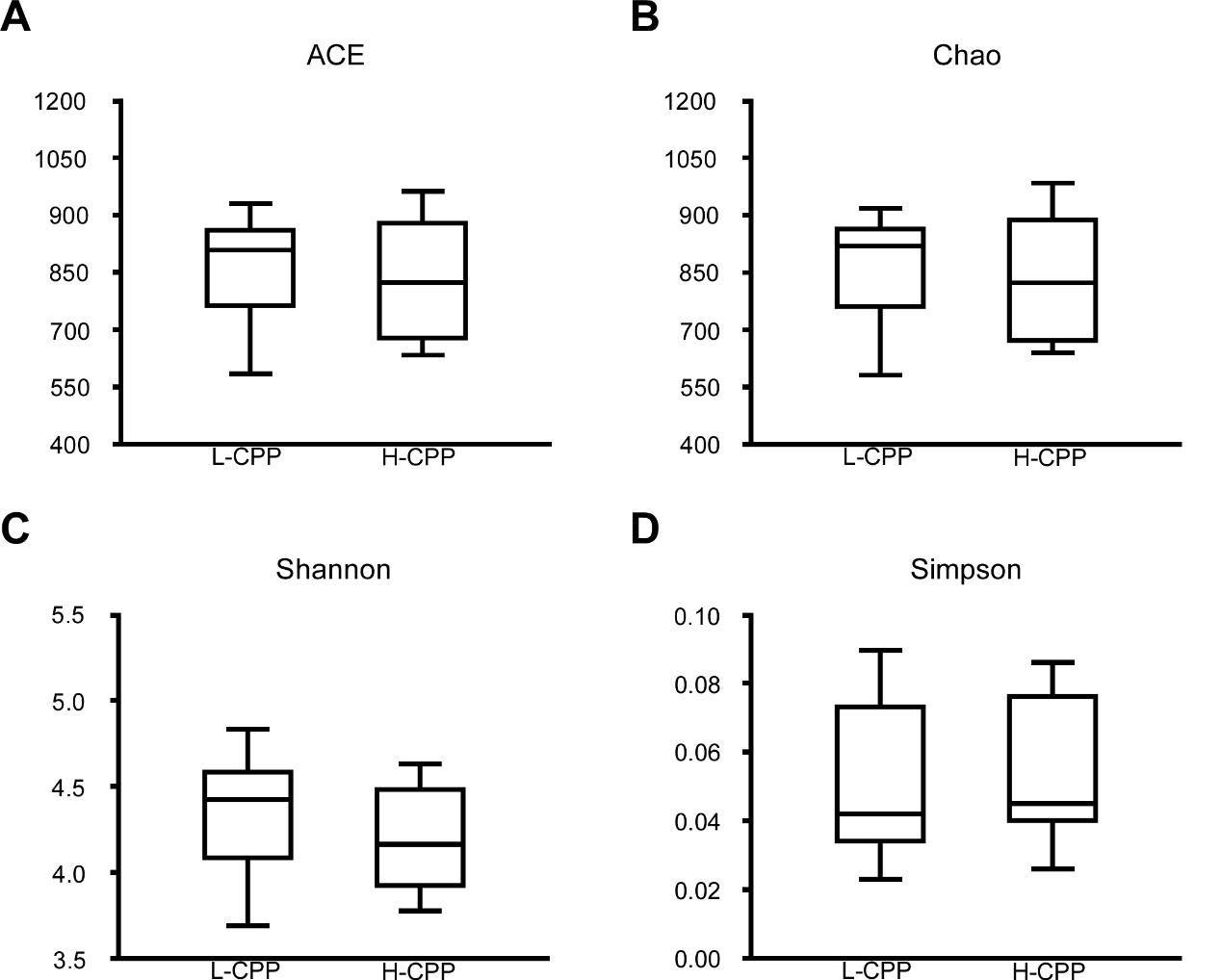


**Figure S2: Comparison of the community alpha diversity between L-CPP and H-CPP.** Alpha diversity was measured by ACE **(A)**, Chao **(B)**, Shannon **(C)** and Simpson **(D)**. A Mann-Whitney test was used to analyze the data. A student’s t test was used for the data with Gaussian distribution. The central line shown in each box plot indicates the median of data. Whiskers extend to cover the whole range of values. Statistical significance was accepted at p<0.05.

1. **Supplementary Tables**

**Table S1:** Gut microbiota analyzed at family and genus levels

| ***Family Acidaminococcaceae*** | ***Family Corynebacteriaceae*** | ***Family Paenibacillaceae_1*** |
| --- | --- | --- |
| ***Family Actinomycetaceae*** | ***Family Deferribacteraceae*** | ***Family Pasteurellaceae*** |
| ***Family Aerococcaceae*** | ***Family Dermabacteraceae*** | ***Family Peptococcaceae_1*** |
| ***Family Alcaligenaceae*** | ***Family Desulfovibrionaceae*** | ***Family Peptostreptococcaceae*** |
| ***Family Anaeroplasmataceae*** | ***Family Dietziaceae*** | ***Family Phyllobacteriaceae*** |
| ***Family Bacillaceae_1*** | ***Family Elusimicrobiaceae*** | ***Family Piscirickettsiaceae*** |
| ***Family Bacillaceae_2*** | ***Family Enterobacteriaceae*** | ***Family Planococcaceae*** |
| ***Family Bacillales_Incertae_Sedis_XI*** | ***Family Enterococcaceae*** | ***Family Porphyromonadaceae*** |
| ***Family Bacteriovoracaceae*** | ***Family Erysipelotrichaceae*** | ***Family Prevotellaceae*** |
| ***Family Bacteroidaceae*** | ***Family Erythrobacteraceae*** | ***Family Promicromonosporaceae*** |
| ***Family Bdellovibrionaceae*** | ***Family Eubacteriaceae*** | ***Family Pseudomonadaceae*** |
| ***Family Bifidobacteriaceae*** | ***Family Flavobacteriaceae*** | ***Family Puniceicoccaceae*** |
| ***Family Bradymonadaceae*** | ***Family Helicobacteraceae*** | ***Family Rhodobacteraceae*** |
| ***Family Bradyrhizobiaceae*** | ***Family Hyphomicrobiaceae*** | ***Family Rhodospirillaceae*** |
| ***Family Brevibacteriaceae*** | ***Family Idiomarinaceae*** | ***Family Rikenellaceae*** |
| ***Family Brucellaceae*** | ***Family Lachnospiraceae*** | ***Family Ruminococcaceae*** |
| ***Family Burkholderiaceae*** | ***Family Lactobacillaceae*** | ***Family Sphingobacteriaceae*** |
| ***Family Carnobacteriaceae*** | ***Family Methanobacteriaceae*** | ***Family Spirochaetaceae*** |
| ***Family Catabacteriaceae*** | ***Family Methanomassiliicoccaceae*** | ***Family Staphylococcaceae*** |
| ***Family Caulobacteraceae*** | ***Family Microbacteriaceae*** | ***Family Streptococcaceae*** |
| ***Family Chloroplast*** | ***Family Micrococcaceae*** | ***Family Sutterellaceae*** |
| ***Family Christensenellaceae*** | ***Family Moraxellaceae*** | ***Family Unassigned*** |
| ***Family Clostridiaceae_1*** | ***Family Mycoplasmataceae*** | ***Family Veillonellaceae*** |
| ***Family Clostridiales_Incertae_Sedis_XIII*** | ***Family Nitrosomonadaceae*** | ***Family Verrucomicrobiaceae*** |
| ***Family Comamonadaceae*** | ***Family No_Rank*** | ***Family Victivallaceae*** |
| ***Family Coriobacteriaceae*** | ***Family Nocardiaceae*** | ***Family Xanthomonadaceae*** |
| ***Genus Acetanaerobacterium*** | ***Genus Coraliomargarita*** | ***Genus Ochrobactrum*** |
| ***Genus Acetatifactor*** | ***Genus Corynebacterium*** | ***Genus Odoribacter*** |
| ***Genus Acinetobacter*** | ***Genus Desulfovibrio*** | ***Genus Okibacterium*** |
| ***Genus Adlercreutzia*** | ***Genus Devosia*** | ***Genus Oligella*** |
| ***Genus Aerococcus*** | ***Genus Dietzia*** | ***Genus Olsenella*** |
| ***Genus Aestuariispira*** | ***Genus Dorea*** | ***Genus Oscillibacter*** |
| ***Genus Akkermansia*** | ***Genus Eisenbergiella*** | ***Genus Paenalcaligenes*** |
| ***Genus Alcaligenes*** | ***Genus Elusimicrobium*** | ***Genus Paenibacillus*** |
| ***Genus Aliidiomarina*** | ***Genus Enteractinococcus*** | ***Genus Paenochrobactrum*** |
| ***Genus Alistipes*** | ***Genus Enterobacter*** | ***Genus Papillibacter*** |
| ***Genus Allobaculum*** | ***Genus Enterococcus*** | ***Genus Parabacteroides*** |
| ***Genus Alloprevotella*** | ***Genus Enterorhabdus*** | ***Genus Paracoccus*** |
| ***Genus Anaerobacterium*** | ***Genus Erysipelotrichaceae_incertae_sedis*** | ***Genus Paraprevotella*** |
| ***Genus Anaerofilum*** | ***Genus Escherichia/Shigella*** | ***Genus Parasutterella*** |
| ***Genus Anaerofustis*** | ***Genus Eubacterium*** | ***Genus Peredibacter*** |
| ***Genus Anaeroplasma*** | ***Genus Facklamia*** | ***Genus Phascolarctobacterium*** |
| ***Genus Anaerorhabdus*** | ***Genus Flaviflexus*** | ***Genus Prevotella*** |
| ***Genus Anaerostipes*** | ***Genus Flavobacterium*** | ***Genus Proteus*** |
| ***Genus Anaerotruncus*** | ***Genus Flavonifractor*** | ***Genus Pseudoflavonifractor*** |
| ***Genus Anaerovorax*** | ***Genus Gemella*** | ***Genus Pseudomonas*** |
| ***Genus Aquamicrobium*** | ***Genus Gemmobacter*** | ***Genus Pseudosphingobacterium*** |
| ***Genus Arthrobacter*** | ***Genus Globicatella*** | ***Genus Psychrobacter*** |
| ***Genus Atopostipes*** | ***Genus Gracilibacillus*** | ***Genus Pusillimonas*** |
| ***Genus Bacillus*** | ***Genus Helicobacter*** | ***Genus Ralstonia*** |
| ***Genus Bacteroides*** | ***Genus Holdemania*** | ***Genus Rhodococcus*** |
| ***Genus Barnesiella*** | ***Genus Intestinimonas*** | ***Genus Rikenella*** |
| ***Genus Bifidobacterium*** | ***Genus Jeotgalicoccus*** | ***Genus Romboutsia*** |
| ***Genus Bilophila*** | ***Genus Kurthia*** | ***Genus Roseburia*** |
| ***Genus Blautia*** | ***Genus Lachnospiracea_incertae_sedis*** | ***Genus Rothia*** |
| ***Genus Bosea*** | ***Genus Lactobacillus*** | ***Genus Ruminococcus*** |
| ***Genus Brachybacterium*** | ***Genus Lactococcus*** | ***Genus Ruminococcus2*** |
| ***Genus Bradymonas*** | ***Genus Leucobacter*** | ***Genus Saccharibacteria_genera_incertae_sedis*** |
| ***Genus Brevibacterium*** | ***Genus Luteimonas*** | ***Genus Schwartzia*** |
| ***Genus Brevundimonas*** | ***Genus Lysinibacillus*** | ***Genus Solibacillus*** |
| ***Genus Butyricicoccus*** | ***Genus Marvinbryantia*** | ***Genus Sphingobacterium*** |
| ***Genus Butyricimonas*** | ***Genus Methanomassiliicoccus*** | ***Genus Sporobacter*** |
| ***Genus Butyrivibrio*** | ***Genus Methanosphaera*** | ***Genus Sporosarcina*** |
| ***Genus Catabacter*** | ***Genus Methylophaga*** | ***Genus Staphylococcus*** |
| ***Genus Cellulosimicrobium*** | ***Genus Microbacterium*** | ***Genus Stenotrophomonas*** |
| ***Genus Cellvibrio*** | ***Genus Moheibacter*** | ***Genus Streptococcus*** |
| ***Genus Christensenella*** | ***Genus Morganella*** | ***Genus Streptophyta*** |
| ***Genus Clostridium_IV*** | ***Genus Mucispirillum*** | ***Genus Subdoligranulum*** |
| ***Genus Clostridium_XlVa*** | ***Genus Murimonas*** | ***Genus Thiopseudomonas*** |
| ***Genus Clostridium_XlVb*** | ***Genus Mycoplasma*** | ***Genus Turicibacter*** |
| ***Genus Clostridium_XVIII*** | ***Genus Myroides*** | ***Genus Unassigned*** |
| ***Genus Collinsella*** | ***Genus Nitrosomonas*** | ***Genus Vagococcus*** |
| ***Genus Comamonas*** | ***Genus No_Rank*** | ***Genus Vampirovibrio*** |
| ***Genus Coprobacillus*** | ***Genus Nosocomiicoccus*** | ***Genus Victivallis*** |
| ***Genus Coprococcus*** | ***Genus Oceanobacillus*** |  |

**Table S2:** Comparison of the gut microbial composition among the baseline and post-treatment for the morphine and saline group at the genus level

| **Genus-taxon** | **Saline-baseline VS Saline** | **Morphine-baseline VS Morphine** | **Saline VS Morphine** |
| --- | --- | --- | --- |
| ***Acetatifactor*** |  | 0.0281 |  |
| ***Adlercreutzia*** | 0.0004 | <0.0001 |  |
| ***Alistipes*** | 0.0032 | <0.0001 |  |
| ***Allobaculum*** |  | 0.0002 |  |
| ***Alloprevotella*** |  | 0.0002 |  |
| ***Anaerofustis*** |  | <0.0001 |  |
| ***Anaerorhabdus*** | 0.0313 | 0.0002 |  |
| ***Anaerovorax*** | 0.0084 | 0.0009 |  |
| ***Bacteroides*** |  | 0.002 |  |
| ***Barnesiella*** |  | <0.0001 |  |
| ***Bilophila*** | 0.0288 | 0.0031 | 0.0287 |
| ***Blautia*** |  | 0.0002 |  |
| ***Butyricicoccus*** |  | 0.034 |  |
| ***Butyricimonas*** | 0.0156 | 0.0012 |  |
| ***Butyrivibrio*** |  | 0.0094 |  |
| ***Catabacter*** | 0.0141 | <0.0001 |  |
| ***Clostridium_XlVa*** | 0.0125 |  | 0.0117 |
| ***Clostridium_XVIII*** | 0.0313 | 0.0004 |  |
| ***Collinsella*** |  | <0.0001 | 0.0045 |
| ***Coprobacillus*** | 0.0156 | 0.0019 |  |
| ***Corynebacterium*** | 0.0156 |  | <0.0001 |
| ***Desulfovibrio*** | 0.0156 | <0.0001 |  |
| ***Enterorhabdus*** |  | 0.0057 |  |
| ***Eisenbergiella*** |  | 0.0116 |  |
| ***Escherichia/Shigella*** |  | 0.0005 | 0.0351 |
| ***Flavonifractor*** |  |  | 0.0402 |
| ***Helicobacter*** |  | 0.011 |  |
| ***Intestinimonas*** | 0.0312 |  |  |
| ***Jeotgalicoccus*** | 0.0156 | 0.0402 | <0.0001 |
| ***Lactococcus*** |  |  | 0.0454 |
| ***Marvinbryantia*** | 0.0313 |  | 0.034 |
| ***Mucispirillum*** | 0.0156 | 0.0022 |  |
| ***Oscillibacter*** | 0.0313 |  |  |
| ***Parasutterella*** | 0.0313 | 0.0061 |  |
| ***Rikenella*** |  | 0.0176 |  |
| ***Romboutsia*** |  | 0.0014 |  |
| ***Roseburia*** |  |  | 0.0104 |
| ***Rothia*** | 0.0156 |  | 0.021 |
| ***Sporobacter*** |  | 0.0025 |  |
| ***Staphylococcus*** |  |  | 0.0401 |
| ***Streptococcus*** | 0.0156 |  | 0.0448 |
| ***Streptophyta*** |  |  | 0.0067 |

A Mann-Whitney test and Wilcoxon signed rank test were used to analyzed the data. Student’s t test was used for data with Gaussian distribution. Values were expressed as p values. Statistical significance was accepted at p<0.05.

**Table S3:** Comparison of the gut microbiome composition among the baseline and post-treatment for the morphine and saline group at the family level

| **Family-taxon** | **Saline-baseline VS Saline** | **Morphine-baseline VS Morphine** | **Saline VS Morphine** |
| --- | --- | --- | --- |
| ***Bacteroidaceae*** |  | 0.0024 |  |
| ***Catabacteriaceae*** | 0.0141 | <0.0001 |  |
| ***Chloroplast*** |  |  | 0.0067 |
| ***Clostridiales_Incertae_Sedis_XIII*** | 0.0084 | 0.0008 |  |
| ***Coriobacteriaceae*** | 0.0181 | 0.0004 |  |
| ***Corynebacteriaceae*** | 0.0156 |  | <0.0001 |
| ***Deferribacteraceae*** | 0.0156 | 0.0022 |  |
| ***Desulfovibrionaceae*** | 0.0093 | 0.0008 |  |
| ***Enterobacteriaceae*** |  | 0.0005 | 0.0309 |
| ***Enterococcaceae*** |  |  | 0.0325 |
| ***Erysipelotrichaceae*** | 0.0156 | 0.0281 |  |
| ***Helicobacteraceae*** |  | 0.011 |  |
| ***Lachnospiraceae*** |  | 0.001 |  |
| ***Microbacteriaceae*** | 0.0243 |  | 0.0025 |
| ***Micrococcaceae*** | 0.0085 |  | 0.0001 |
| ***No_Rank*** |  | 0.0402 |  |
| ***Pasteurellaceae*** |  | <0.0001 | 0.002 |
| ***Peptococcaceae_1*** | 0.0156 | 0.0034 |  |
| ***Peptostreptococcaceae*** |  | 0.0014 |  |
| ***Puniceicoccaceae*** |  | 0.0002 |  |
| ***Rikenellaceae*** | 0.0156 | <0.0001 |  |
| ***Spirochaetaceae*** | 0.0156 | 0.0006 |  |
| ***Staphylococcaceae*** |  |  | 0.0035 |
| ***Streptococcaceae*** | 0.0156 |  | 0.0201 |
| ***Sutterellaceae*** | 0.0313 | 0.0061 |  |

Mann-Whitney test and Wilcoxon signed rank test were used to analyzed the data. Student’s t test was used for data with Gaussian distribution. Values were expressed as p values. Statistical significance was accepted at p<0.05.

**Table S4:** Comparison of the gut microbial H-CPP and L-CPP group after the morphine-induced CPP training both at the genus and the family levels

| **Taxon** | **L-CPP VS H-CPP** |
| --- | --- |
| ***Family Lachnospiraceae*** | 0.008 |
| ***Family Ruminococcaceae*** | 0.0066 |
| ***Family Porphyromonadaceae*** | 0.0023 |
| ***Family Peptostreptococcaceae*** | 0.01 |
| ***Family Spirochaetaceae*** | 0.0252 |
| ***Family Veillonellaceae*** | 0.0236 |
| ***Family Catabacteriaceae*** | 0.0087 |
| ***Family Elusimicrobiaceae*** | 0.0196 |
| ***Family Christensenellaceae*** | 0.0474 |
| ***Genus Alloprevotella*** | 0.0384 |
| ***Genus Romboutsia*** | 0.01 |
| ***Genus Clostridium_IV*** | 0.0387 |
| ***Genus Roseburia*** | 0.0036 |
| ***Genus Schwartzia*** | 0.0236 |
| ***Genus Catabacter*** | 0.0087 |
| ***Genus Elusimicrobium*** | 0.0196 |
| ***Genus Dorea*** | 0.0423 |
| ***Genus Christensenella*** | 0.0471 |
| ***Genus Anaerofilum*** | 0.0185 |

A Mann-Whitney test was used to analyze the data. A student’s t test was used for the data with Gaussian distribution. Values were expressed as p values. Statistical significance was accepted at p<0.05.

**Table S5:** Comparison of the within-group differences in the gut microbiome composition for L-CPP and H-CPP groups between the pre- and post-conditioning at the genus level

| **Genus-taxon** | **L-CPP-baseline VS L-CPP** | **H-CPP-baseline VS H-CPP** |
| --- | --- | --- |
| ***Butyricicoccus*** | 0.0244 |  |
| ***Rikenella*** | 0.0093 |  |
| ***Clostridium_XVIII*** | 0.002 |  |
| ***Sporobacter*** | 0.0264 |  |
| ***Coprobacillus*** | 0.0112 |  |
| ***Anaerorhabdus*** | 0.0015 |  |
| ***Enterococcus*** | 0.0469 |  |
| ***Romboutsia*** |  | 0.0049 |
| ***Parasutterella*** |  | 0.0425 |
| ***Bilophila*** |  | 0.0254 |
| ***Mucispirillum*** |  | 0.0039 |
| ***Enterorhabdus*** |  | 0.0244 |
| ***Rothia*** |  | 0.0189 |
| ***Bacteroides*** |  | 0.0269 |
| ***Anaerovorax*** |  | 0.0396 |
| ***Saccharibacteria_genera_incertae_sedis*** |  | 0.0204 |
| ***Alloprevotella*** | 0.0032 | 0.0486 |
| ***Barnesiella*** | 0.0006 | 0.0122 |
| ***Blautia*** | 0.0342 | 0.0015 |
| ***Escherichia/Shigella*** | 0.0186 | 0.0068 |
| ***Desulfovibrio*** | 0.002 | 0.002 |
| ***Catabacter*** | 0.0195 | 0.0041 |
| ***Collinsella*** | 0.0439 | 0.002 |
| ***Alistipes*** | 0.0039 | 0.024 |
| ***Adlercreutzia*** | 0.0149 | 0.0024 |

A Wilcoxon signed rank test was used to analyzed the data. A student’s t test was used for data with Gaussian distribution. Values were expressed as p values. Statistical significance was accepted at p<0.05.

**Table S6:** Comparison of the within-group differences in the gut microbiome composition for L-CPP and H-CPP groups between the pre- and post-conditioning at the family level.

| **Family-taxon** | **L-CPP-baseline VS L-CPP** | **H-CPP-baseline VS H-CPP** |
| --- | --- | --- |
| ***Peptococcaceae_1*** | 0.0075 |  |
| ***Lachnospiraceae*** | 0.0042 |  |
| ***Ruminococcaceae*** | 0.0342 |  |
| ***Porphyromonadaceae*** | 0.0093 |  |
| ***Puniceicoccaceae*** | 0.0039 |  |
| ***Erysipelotrichaceae*** |  | 0.0425 |
| ***Peptostreptococcaceae*** |  | 0.0049 |
| ***Sutterellaceae*** |  | 0.0425 |
| ***Desulfovibrionaceae*** |  | 0.0034 |
| ***Staphylococcaceae*** |  | 0.0425 |
| ***Deferribacteraceae*** |  | 0.0039 |
| ***Bacteroidaceae*** |  | 0.0269 |
| ***Spirochaetaceae*** |  | 0.001 |
| ***Clostridiales_Incertae_Sedis_XIII*** |  | 0.0358 |
| ***Catabacteriaceae*** | 0.0195 | 0.0041 |
| ***Enterobacteriaceae*** | 0.0137 | 0.0068 |
| ***Pasteurellaceae*** | 0.001 | 0.0059 |
| ***Coriobacteriaceae*** | 0.0034 | 0.042 |
| ***Rikenellaceae*** | 0.0025 | 0.0196 |

A Wilcoxon signed rank test was used to analyzed the data. A student’s t test was used for data with Gaussian distribution. Values were expressed as p values. Statistical significance was accepted at p<0.05.
